# Supplementary material for: Genetic and Phylogenetic Analysis of Feline Coronavirus in Guangxi Province of China from 2021 to 2024
Source: Vet Sci. 2024 Sep 25;11(10):455. doi: 10.3390/vetsci11100455 (PMC11512343; doi:10.3390/vetsci11100455)
Supplement: Supplementary file 1 [file vetsci-11-00455-s001.zip › vetsci-3187566-supplementary.pdf]

Supplementary Materials

Table S1. The information on the FCoV gene sequences obtained in this study.

Host	Time	Location	Sample Type	Strain	Gene Accession Number		
					S	M	N
Feline	Jul, 2023	Beihai	feces	GXBH01-2023	PP448795	PP444724	PP448735
Feline	May, 2022	Baise	anal swab	GXBS01-2022	PP448796	PP444725	PP448736
Feline	May, 2022	Baise	ascites	GXBS02-2022	PP448797	PP444726	PP448737
Feline	May, 2022	Baise	ascites	GXBS03-2022	PP448798	PP444727	PP448738
Feline	Sep, 2022	Baise	anal swab	GXBS04-2022	PP448799	PP444728	PP448739
Feline	Oct, 2022	Baise	anal and nasal swabs	GXBS05-2022	PP448800	PP444729	PP448740
Feline	Jan, 2023	Baise	anal and nasal swabs	GXBS06-2023	PP448801	PP444730	PP448741
Feline	Mar, 2023	Baise	anal swab	GXBS07-2023	PP448802	PP444731	PP448742
Feline	Mar, 2023	Baise	anal and nasal swabs	GXBS08-2023	PP448803	PP444732	PP448743
Feline	May, 2023	Baise	anal swab	GXBS09-2023	PP448804	PP444733	PP448744
Feline	Nov, 2023	Baise	anal swab	GXBS10-2023	PP448805	PP444734	PP448745
Feline	Nov, 2023	Baise	anal and nasal swabs	GXBS11-2023	PP448806	PP444735	PP448746
Feline	Oct, 2022	Guilin	ascites	GXGL01-2022	PP448807	PP444736	PP448747
Feline	Oct, 2022	Guilin	ascites	GXGL02-2022	PP448808	PP444737	PP448748
Feline	May, 2023	Guilin	anal swab	GXGL03-2023	PP448809	PP444738	PP448749
Feline	Jun, 2023	Guilin	anal swab	GXGL04-2023	PP448810	PP444739	PP448750
Feline	Nov, 2023	Guilin	anal and nasal swabs	GXGL05-2023	PP448811	PP444740	PP448751
Feline	Nov, 2023	Guilin	anal and nasal swabs	GXGL06-2023	PP448812	PP444741	PP448752
Feline	Dec, 2021	Hechi	anal swab	GXHC01-2021	PP448813	PP444742	PP448753
Feline	Dec, 2021	Hechi	anal swab	GXHC02-2021	PP448814	PP444743	PP448754
Feline	Dec, 2021	Hechi	anal swab	GXHC03-2021	PP448815	PP444744	PP448755
Feline	Mar, 2022	Liuzhou	feces	GXLZ01-2022	PP448816	PP444745	PP448756
Feline	Mar, 2022	Liuzhou	feces	GXLZ02-2022	PP448817	PP444746	PP448757
Feline	Apr, 2022	Liuzhou	nasal swab	GXLZ03-2022	PP448818	PP444747	PP448758
Feline	Mar, 2022	Liuzhou	feces	GXLZ04-2022	PP448819	PP444748	PP448759
Feline	Apr, 2022	Liuzhou	feces	GXLZ05-2022	PP448820	PP444749	PP448760
Feline	Apr, 2022	Liuzhou	anal swab	GXLZ06-2022	PP448821	PP444750	PP448761
Feline	Jun, 2022	Liuzhou	feces	GXLZ07-2022	PP448822	PP444751	PP448762
Feline	May, 2022	Liuzhou	nasal swab	GXLZ08-2022	PP448823	PP444752	PP448763
Feline	May, 2022	Liuzhou	feces	GXLZ09-2022	PP448824	PP444753	PP448764
Feline	May, 2022	Liuzhou	anal swab	GXLZ10-2022	PP448825	PP444754	PP448765
Feline	May, 2022	Liuzhou	feces	GXLZ11-2022	PP448826	PP444755	PP448766
Feline	Jun, 2022	Liuzhou	feces	GXLZ12-2022	PP448827	PP444756	PP448767
Feline	Jun, 2022	Liuzhou	feces	GXLZ13-2022	PP448828	PP444757	PP448768
Feline	Aug, 2022	Liuzhou	feces	GXLZ14-2022	PP448829	PP444758	PP448769
Feline	Oct, 2022	Liuzhou	feces	GXLZ15-2022	PP448830	PP444759	PP448770
Feline	Mar, 2023	Liuzhou	anal swab	GXLZ16-2023	PP448831	PP444760	PP448771

Feline	Jul, 2023	Liuzhou	ascites	GXLZ17-2023	PP448832	PP444761	PP448772
Feline	Sep, 2023	Liuzhou	feces	GXLZ18-2023	PP448833	PP444762	PP448773
Feline	Nov, 2023	Liuzhou	feces	GXLZ19-2023	PP448834	PP444763	PP448774
Feline	Sep, 2023	Liuzhou	feces	GXLZ20-2023	PP448835	PP444764	PP448775
Feline	Apr, 2022	Nanning	anal swab	GXNN01-2022	PP448836	PP444765	PP448776
Feline	May, 2022	Nanning	feces	GXNN02-2022	PP448837	PP444766	PP448777
Feline	Jun, 2022	Nanning	feces	GXNN03-2022	PP448838	PP444767	PP448778
Feline	May, 2022	Nanning	anal swab	GXNN04-2022	PP448839	PP444768	PP448779
Feline	Sep, 2022	Nanning	nasal swab	GXNN05-2022	PP448840	PP444769	PP448780
Feline	Oct, 2022	Nanning	anal and nasal swabs	GXNN06-2022	PP448841	PP444770	PP448781
Feline	Jan, 2023	Nanning	feces	GXNN07-2023	PP448842	PP444771	PP448782
Feline	Apr, 2023	Nanning	anal and nasal swabs	GXNN08-2023	PP448843	PP444772	PP448783
Feline	May, 2023	Nanning	feces	GXNN09-2023	PP448844	PP444773	PP448784
Feline	Oct, 2023	Nanning	feces	GXNN10-2023	PP448845	PP444774	PP448785
Feline	Aug, 2023	Nanning	anal and nasal swabs	GXNN11-2023	PP448846	PP444775	PP448786
Feline	Jun, 2023	Qinzhou	anal and nasal swabs	GXQZ01-2023	PP448847	PP444776	PP448787
Feline	Aug, 2023	Qinzhou	anal and nasal swabs	GXQZ02-2023	PP448848	PP444777	PP448788
Feline	Dec, 2023	Qinzhou	anal and nasal swabs	GXQZ03-2023	PP448849	PP444778	PP448789
Feline	Jun, 2022	Yulin	anal swab	GXYL01-2022	PP448850	PP444779	PP448790
Feline	Jun, 2022	Yulin	anal swab	GXYL02-2022	PP448851	PP444780	PP448791
Feline	Aug, 2022	Yulin	feces	GXYL03-2022	PP448852	PP444781	PP448792
Feline	Nov, 2022	Yulin	anal swab	GXYL04-2022	PP448853	PP444782	PP448793
Feline	Nov, 2022	Yulin	feces	GXYL05-2022	PP448854	PP444783	PP448794
Feline	Dec, 2023	Beihai	feces	GXBH02-2024	PP464266	PP464260	PP464263
Feline	Mar, 2024	Qinzhou	feces	GXQZ04-2024	PP464267	PP464261	PP464264
Feline	Feb, 2024	Qinzhou	feces	GXQZ05-2024	PP464268	PP464262	PP464265

Table S2. The information on the referenced FCoV (FECV, FIPV), CCoV, and TGEV used in this study

Strain	Location	Code Letter	Date	Host	Gene Accession Number			Source
					S	M	N	
FECV MY0622	China: Sichuan	CHN	2020	Feline	MW815650	MW722859	MW722825	GenBank
FECV LS0610	China: Sichuan	CHN	2020	Feline	MW815651	MW722860	MW722826	GenBank
FECV CD0610	China: Sichuan	CHN	2020	Feline	MW815652	MW722861	MW722827	GenBank
FECV CD0616-1	China: Sichuan	CHN	2020	Feline	MW815653	MW722862	MW722828	GenBank
FECV CD0616-2	China: Sichuan	CHN	2020	Feline	MW815654	MW722863	MW722829	GenBank
FECV CD0617	China: Sichuan	CHN	2020	Feline	MW815655	MW722864	MW722830	GenBank
FECV CD0523	China: Sichuan	CHN	2020	Feline	MW815656	MW722865	MW722831	GenBank
FECV CD0524	China: Sichuan	CHN	2020	Feline	MW815657	MW722866	MW722832	GenBank
FECV LS0612	China: Sichuan	CHN	2020	Feline	MW815658	MW722867	MW722833	GenBank
FECV DY0615	China: Sichuan	CHN	2020	Feline	MW815659	MW722868	MW722834	GenBank
FECV CD0607	China: Sichuan	CHN	2020	Feline	MW815660	MW722869	MW722835	GenBank
FECV CD0521	China: Sichuan	CHN	2020	Feline	MW815661	MW722870	MW722836	GenBank
FECV SN0623	China: Sichuan	CHN	2020	Feline	MW815662	MW722871	MW722837	GenBank
FIPV GY0528	China: Sichuan	CHN	2020	Feline	/	MW722872	MW722838	GenBank
FIPV NC0521	China: Sichuan	CHN	2020	Feline	/	MW722873	MW722839	GenBank
FIPV MY0628	China: Sichuan	CHN	2020	Feline	/	MW722874	MW722840	GenBank
FIPV CD0402	China: Sichuan	CHN	2020	Feline	/	MW722875	MW722841	GenBank
FIPV CD0522	China: Sichuan	CHN	2020	Feline	/	MW722876	MW722842	GenBank
FIPV 79-1146	The United States	USA	1979	Feline	AY994055，complete genome			GenBank
FIPV C1Je	United Kingdom	UK	2006	Feline	DQ848678，complete genome			GenBank
FIPV Black	The United States	USA	1975	Feline	EU186072，complete genome			GenBank
FCoV RM	USA: California	USA	2002	Feline	FJ938051，complete genome			GenBank
FCoV UU11	The Netherlands	NED	2007	Feline	FJ938052，complete genome			GenBank
FCoV UU7	The Netherlands	NED	2007	Feline	FJ938053，complete genome			GenBank
FIPV UU4	The Netherlands	NED	2007	Feline	FJ938054，complete genome			GenBank
FIPV UU8	The Netherlands	NED	2007	Feline	FJ938055，complete genome			GenBank
FIPV UU5	The Netherlands	NED	2007	Feline	FJ938056，complete genome			GenBank
FIPV UU15	The Netherlands	NED	2007	Feline	FJ938057，complete genome			GenBank
FIPV UU16	The Netherlands	NED	2007	Feline	FJ938058，complete genome			GenBank
FCoV UU10	The Netherlands	NED	2007	Feline	FJ938059，complete genome			GenBank
FCoV UU2	USA: California	USA	1993	Feline	FJ938060，complete genome			GenBank
FIPV UU3	USA: California	USA	1998	Feline	FJ938061，complete genome			GenBank
FIPV UU9	The Netherlands	NED	2007	Feline	FJ938062，complete genome			GenBank
FCoV/NTU156/P	China: Taiwan	CHN_TW	2007	Feline	GQ152141，complete genome			GenBank
FCoV UU22	The Netherlands	NED	2007	Feline	GU553361，complete genome			GenBank
FCoV UU23	The Netherlands	NED	2007	Feline	GU553362，complete genome			GenBank
FIPV UU17	The Netherlands	NED	2007	Feline	HQ012367，complete genome			GenBank
FCoV UU18	The Netherlands	NED	2007	Feline	HQ012368，complete genome			GenBank
FIPV UU21	The Netherlands	NED	2007	Feline	HQ012369，complete genome			GenBank

Strain	Location	Code Letter	Date	Host	Gene Accession Number			Source
					S	M	N	
FIPV UU24	The Netherlands	NED	2008	Feline		HQ012370，complete genome		GenBank
FCoV UU31	The Netherlands	NED	2008	Feline		HQ012371，complete genome		GenBank
FIPV UU30	The Netherlands	NED	2008	Feline		HQ392472，complete genome		GenBank
FCoV UU47	The Netherlands	NED	2010	Feline		JN183882，complete genome		GenBank
FCoV UU54	The Netherlands	NED	2010	Feline		JN183883，complete genome		GenBank
FIPV DF-2	The United States	USA	1980	Feline		JQ408981，complete genome		GenBank
FCoV UU88	The Netherlands	NED	2010	Feline		KF530123，complete genome		GenBank
FIPV isolate 27C	United Kingdom	UK	2011	Feline		KP143507，complete genome		GenBank
FIPV isolate 28O	United Kingdom	UK	2011	Feline		KP143508，complete genome		GenBank
FCoV isolate 65F	United Kingdom	UK	2011	Feline		KP143509，complete genome		GenBank
FCoV isolate 67F	United Kingdom	UK	2011	Feline		KP143510，complete genome		GenBank
FCoV isolate 80F	United Kingdom	UK	2011	Feline		KP143511，complete genome		GenBank
FIPV isolate 26M	United Kingdom	UK	2011	Feline		KP143512，complete genome		GenBank
FCoV inoculum	Belgium	BEL	2013	Feline		KU215419，complete genome		GenBank
FCoV Cat1_day7	Belgium	BEL	2013	Feline		KU215420，complete genome		GenBank
Cat2_day21_deletion	Belgium	BEL	2013	Feline		KU215421，complete genome		GenBank
FCoV Cat2_day21_w_d	Belgium	BEL	2013	Feline		KU215422，complete genome		GenBank
FCoV Cat3_day9	Belgium	BEL	2013	Feline		KU215423，complete genome		GenBank
FCoV Cat1_day28_d	Belgium	BEL	2013	Feline		KU215424，complete genome		GenBank
FCoV Cat1day28_ w_d	Belgium	BEL	2013	Feline		KU215425，complete genome		GenBank
FCoV UG-FH8	Belgium	BEL	2015	Feline		KX722529，complete genome		GenBank
FIPV Cat 1 Karlslunde	Denmark	DEN	2015	Feline		KX722530，complete genome		GenBank
FIPV HLJ/DQ/2016/01	China	CHN	2016	Feline		KY292377，complete genome		GenBank
FIPV HLJ/HRB/2016/10	China	CHN	2016	Feline		KY566209，complete genome		GenBank
FIPV HLJ/HRB/2016/11	China	CHN	2016	Feline		KY566210，complete genome		GenBank
FIPV HLJ/HRB/2016/13	China	CHN	2016	Feline		KY566211，complete genome		GenBank
FCoV Felix	Germany	GER	2012	Feline		MG893511，complete genome		GenBank
FCoV isolate XXN	China	CHN	2018	Feline		MN165107，complete genome		GenBank
FIPV ZJU1617	China: Zhejiang	CHN	2016	Feline		MT239439，complete genome		GenBank
FIPV ZJU1709	China: Zhejiang	CHN	2017	Feline		MT239440，complete genome		GenBank
FIPV HF1902	China	CHN	2019	Feline		MT444152，complete genome		GenBank
FCoV isolate QS	China	CHN	2018	Feline		MW030108，complete genome		GenBank
FCoV isolate SD	China	CHN	2018	Feline		MW030110，complete genome		GenBank
FCoV VP1a	Germany	GER	2006	Feline		MW308128，complete genome		GenBank
FCoV_JP15_Fe_35_2015	Japan	JPN	2015	Feline		LC742526，complete genome		GenBank
FCoV HC-8	Australia	AUS	2018	Feline		ON595869，complete genome		GenBank
FCoV SB22	Brazil	BRA	2015	Feline		MH817484，complete genome		GenBank
FIPV SH2021	China	CHN	2021	Feline		OR295209，complete genome		GenBank
FCoV HC-1	Australia	AUS	2018	Feline		ON595862，complete genome		GenBank
FCoV 2020-FM3	The United States	USA	2020	Feline		OP542206，complete genome		GenBank
FCoV FPV-3	Australia	AUS	2017	Feline		ON595855，complete genome		GenBank
FCoV FPV-6	Australia	AUS	2017	Feline		ON595858，complete genome		GenBank

Strain	Location	Code Letter	Date	Host	Gene Accession Number			Source
					S	M	N	
FCoV FPV-4	Australia	AUS	2017	Feline	ON595856，complete genome			GenBank
FCoV HC-10	Australia	AUS	2018	Feline	ON595871，complete genome			GenBank
FCoV FPV-5	Australia	AUS	2017	Feline	ON595857，complete genome			GenBank
FCoV Cat 2 Holstebro	Denmark	DEN	2015	Feline	KX722531，complete genome			GenBank
FCoV OH11927	USA: Ohio	USA	2014	Feline	MF457591，complete genome			GenBank
FCoV Tokyo/cat/130627	Japan:Tokyo	JPN	2013	Feline	AB907624，S M N gene CDs			GenBank
FCoV/NTU2/R/2003	China: Taiwan	CHN_TW	2003	Feline	DQ160294，S M N gene CDs			GenBank
FCoV C3663	Japan	JPN	1994	Feline	AB535528，S M Ngene CDs			GenBank
FIPV UCD1	The United States	USA	1976	Feline	AB088222，S gene CDs			GenBank
FCoV Yayoi	Japan: Tokyo	JPN	1991	Feline	AB695067，S gene CDs			GenBank
FCoV M91-267	Japan	JPN	1991	Feline	AB781788，S gene CDs			GenBank
FCoV KUK-H/L	Japan	JPN	1987	Feline	AB781789，S gene CDs			GenBank
FCoV KU-2	Japan	JPN	1991	Feline	D32044，S gene CDs			GenBank
FIPV-UCD11a	The United States	USA	2008	Feline	FJ917519，S gene CDs			GenBank
FIPV-UCD11b	The United States	USA	2008	Feline	FJ917520，S gene CDs			GenBank
FIPV-UCD12	The United States	USA	2008	Feline	FJ917521，S gene CDs			GenBank
FECV-UCD5	The United States	USA	2008	Feline	FJ917522，S gene CDs			GenBank
FCoV HRB/XF17	China	CHN	2017	Feline	MK987175，S gene CDs			GenBank
FCoV SMU CD77	China	CHN	2019	Feline	MW316839，S gene CDs			GenBank
FCoV SMU CQ86	China	CHN	2019	Feline	MW316840，S gene CDs			GenBank
FCoV SMU CDF12	China	CHN	2020	Feline	MW316841，S gene CDs			GenBank
FCoV SMU CDF97	China	CHN	2020	Feline	MW316847，S gene CDs			GenBank
FCoV F21061627-1	China	CHN	2021	Feline	OQ351917，S gene CDs			GenBank
FCoV F21071412-2	China	CHN	2021	Feline	OQ351918，S gene CDs			GenBank
FCoV SMU CD61	China	CHN	2018	Feline	MW316852，S gene CDs			GenBank
FCoV SMU-CD59	China	CHN	2018	Feline	MW316851，S gene CDs			GenBank
FCoV SMU-CQ18	China	CHN	2018	Feline	MW316850，S gene CDs			GenBank
FCoV local7_C4	Brazil	BRA	2011	Feline	KJ879408，M gene CDs			GenBank
FCoV local7_C60	Brazil	BRA	2011	Feline	KJ879405，M gene CDs			GenBank
FCoV local3_C27	Brazil	BRA	2011	Feline	KJ879402，M gene CDs			GenBank
FCoV local2_C27	Brazil	BRA	2012	Feline	KJ879397，M gene CDs			GenBank
FCoV BJ_2016_01	China	CHN	2016	Feline	KY566205，N gene CDs			GenBank
FCoV HLJ_DQ_2016_01-d29	China	CHN	2016	Feline	KY587362，N gene CDs			GenBank
FCoV TN_369_00	Italy	ITA	2000	Feline	GU017126，N gene CDs			GenBank
FCoV TN_376_00	Italy	ITA	2000	Feline	GU017125，N gene CDs			GenBank
FCoV PC_Bimba_05	Italy	ITA	2005	Feline	GU017121，N gene CDs			GenBank
FCoV MI_cat8_04	Italy	ITA	2004	Feline	GU017120，N gene CDs			GenBank
CCoV 23/03	Italy	ITA	2003	Canine	KP849472，complete genome			GenBank
CCoV 10/22	United Kingdom	UK	2022	Canine	OX335534，complete genome			GenBank
CCoV 12/20	United Kingdom	UK	2022	Canine	OX335549，complete genome			GenBank
CCoV 2020/15	United Kingdom	UK	2020	Canine	MT906864，complete genome			GenBank

Strain	Location	Code Letter	Date	Host	Gene Accession Number			Source
					S	M	N	
CCoV A76	USA: Ithaca, NY	USA	1976	Canine	JN856008，complete genome			GenBank
CCoV 341/05	Italy	ITA	2005	Canine	EU856361，complete genome			GenBank
CCoV 68/09	Greece	GRC	2009	Canine	HQ450377，complete genome			GenBank
CCoV/GD/2020/X9	China	CHN	2020	Canine	MZ320954，complete genome			GenBank
CCoV B135_JS_2018	China	CHN	2019	Canine	MT114544，complete genome			GenBank
CCoV HLJ-071	China	CHN	2016	Canine	KY063616，complete genome			GenBank
TGEV SC2021	China	CHN	2021	Swine	ON858825，complete genome			GenBank
TGEV CH8438	China	CHN	2017	Swine	MW804449，complete genome			GenBank
TGEV HB-1	China	CHN	2020	Swine	MZ368889，complete genome			GenBank
TGEV 154_2014	The United States	USA	2014	Swine	KX900411，complete genome			GenBank
TGEV 153_2014	The United States	USA	2014	Swine	KX900410，complete genome			GenBank
TGEV 145/2008	Mexico	MEX	2008	Swine	KX900402，complete genome			GenBank
TGEV USA Z 1986	The United States	USA	2006	Swine	KX900393，complete genome			GenBank
TGEV HE-1	China	CHN	2015	Swine	KX083668，complete genome			GenBank
